# Stakeholder views on continuing professional development for doctors working in public primary care facilities in central Uganda: a qualitative study

**DOI:** 10.11604/pamj.2024.47.97.417840

**Published:** 2024-02-29

**Authors:** Jane Frances Namatovu, Aloysius Gonzaga Mubuuke, William Buwembo, Janet Nakigudde, Sarah Kiguli

**Affiliations:** 1 Department of Family Medicine, Makerere University, Kampala, Uganda,; 2 Department of Radiology, Makerere University, Kampala, Uganda,; 3 Department of Anatomy, Makerere University, Kampala, Uganda,; 4 Department of Psychiatry, Makerere University, Kampala, Uganda,; 5 Department of Peadiatrics and Child Health, Makerere University, Kampala, Uganda

**Keywords:** Stakeholders, primary care doctors, professional development, district health system

## Abstract

**Introduction:**

the primary care workforce in the public sector of Uganda is under the district health system. The doctors in this workforce provide leadership and frontline promotive, preventive, curative, rehabilitative, and palliative care. Their numbers are still low and therefore need effective support through continuing professional development (CPD). Part of the support is influenced by stakeholders whose views on CPD in the district health system are important. This study therefore explored the stakeholders’ views on the CPD of doctors working in the district health system in central Uganda.

**Methods:**

a qualitative exploratory study was done, and data was collected using an interview guide through in-depth interviews among ten purposively selected CPD stakeholders influencing different aspects of CPD activities of doctors working in public general hospitals and health center IVs. The interviews were recorded and transcribed verbatim and manually analyzed using deductive thematic analysis.

**Results:**

five themes were categorized into; CPD practices, facilitators, benefits, challenges, and suggestions. Each of the themes had subthemes; CPD practices; training, mentorship and apprenticeship, support supervision, and quality improvement projects. Facilitators; internet services, grants, health facility managers, facility-based CPD providers, and regional CPD guidelines. Benefits; motivation, knowledge, teamwork, and renewal of practicing licenses. Challenges; workload, allowances, access, documentation, mindset, quality, structure of public health system, and sustainability. Suggestions; training needs analysis, collaboration, monitoring, e-CPD platforms, CPD resource centers, and individual CPD responsibility.

**Conclusion:**

the stakeholders’ views are an indication that effective CPD is a collaborative effort from both the primary care doctors and those in the leadership of the health care system.

## Introduction

Continuing professional development (CPD) refers to the learning activities that professionals engage in to develop and enhance their abilities and competencies [1] and it is a commitment to ongoing lifelong learning. The World Health Organization (WHO) report of the global conference on primary healthcare emphasizes the need for educating and scaling up the primary healthcare workforce to achieve universal health coverage [2]. One of the recommended ways is through enhancing the skills of primary care doctors to ensure that they can deliver comprehensive services to the community. This in the long run results in quality primary care services for individuals, families, and communities advocated by the Astana Declaration [3]. The emergency of challenges in primary health care calls for appropriate practical solutions that can be well articulated during CPD. Before proposing a revised approach to CPD however, it is important to understand the current practices. This is likely to assist in the assessment of predicted future requirements, encourage long-term preparedness, and anticipate the future of CPD. There is evidence about the impact of CPD including; knowledge, practice and attitude change, career development, and networking among others [4-7].

The status of CPD for different health cadres is at different levels in different countries. For instance, in Uganda, efforts to investigate the CPD training needs of doctors in public primary care facilities have been conducted. Important to note is that in most African countries, doctors are expected to have evidence of CPD before renewal of their annual practicing licenses. The lack of emphasis on mandatory CPD could result in a lack of recognition by other professional bodies across borders, a lack of protection against litigation when the knowledge and skills of doctors are challenged in the courts of law, and a lack of professional practice for patients [8,9]. This is contrary to some developed countries, where CPD for doctors has evolved from an organizational to an individual responsibility [10,11].

Examples of CPD for primary care doctors in Africa include; physical sessions by the CPD Unit of the Department of Family Medicine at the University of Pretoria [12], online sessions by; the South African Academy of Family Physicians (SAAFP) [13], and African forum for PHC (AfroPHC) in partnership with World Continuing Education Alliance (WCEA) [14]. A description of CPD for the different primary care providers in the health care system has also been reported for Malawi and Tanzania [15] The primary healthcare workforce in the public sector of Uganda is organized under the district health system (DHS). The doctors are still few and therefore need effective support through CPD to execute their various duties, especially in rural areas. This CPD support is controlled by stakeholders whose influence determines the success of CPD. These include; the regulators, the CPD providers, and the healthcare system managers. The stakeholders also contribute to the same mandate of ensuring quality healthcare services to the population they serve. Currently, like in many low-income countries, little is known about the views of the key stakeholders affected by the current organization of CPD for primary care doctors in the DHS in Uganda. The purpose of this study therefore was to explore the stakeholders´ views on CPD for doctors working in the DHS in central Uganda. Findings from the study may potentially be useful in many other low-resource settings where the scarcity of primary care doctors especially in underserved rural communities is still prevalent.

## Methods

**Study design:** it was a qualitative exploratory study conducted through individual in-depth interviews with key CPD stakeholders in the central region of the Ugandan public health care system.

**Setting:** the study was conducted in central Uganda. In Uganda, it is mandatory to have, as a requirement for renewal of a doctor´s annual practicing license, evidence of CPD. This is per the Uganda Medical and Dental Practitioners Act of 1996. This Act has so far evolved into the revised CPD accreditation and certification guidelines that have been in use since 2017 to date. Uganda's public health care system consists of the Central Ministry of Health and the DHS. The Ministry of Health is the overall supervisor of the national health system while the DHS is responsible for the provision of primary care services. These services start with Health Center I (VHT) to Health Center IV (HC IV) as well as the general hospitals (GH). The DHS is under the stewardship of the District Health Officer (DHO) deployed by the local government. Each district has at least a HC IV while the GH usually serves a district or a combination of districts. According to the 2016 Uganda Ministry of Health guidelines to the local government planning process health sector supplement, the GH and HC IV have staffing norms of up to six and two primary care doctors respectively.

**Selection of participants:** the key CPD stakeholder categories were identified in central Uganda to participate in the study. Participants were purposively selected to include CPD stakeholders influencing different aspects of CPD activities of doctors working in public GHs and HC IVs. These included; the Uganda Medical Association (UMA)-central branch (this body is interested in the professional growth of its members and it often gives continuing medical education sessions), Uganda Medical and Dental Practitioners Council (UMDPC) (this is the CPD regulatory body in Uganda, for doctors), District Health Officers (administrative supervisors of the doctors) and medical superintendents (these are immediate and clinical supervisors). The selected participants were contacted and invited to participate by email or phone one after another. Interviews were conducted from June 2022 to February 2023. Interviewing was stopped when no new information was being generated from the last three interviews and when it was deemed saturation which had been achieved.

**Data collection:** the principal investigator (PI) and a research assistant (RA) experienced in qualitative designs and data processes conducted the in-depth interviews using an interview guide (Table 1). The PI and RA asked the selected participants to reflect on what is happening with CPD for doctors working in public GHs and HC IVs in the central region of Uganda. Interviews were conducted in English and audiotaped using a digital voice recording application on a smartphone. Interviews lasted 30 to 40 minutes.

**Table 1:** semi-structured interview guide

Stakeholder views on CPD for doctors working in public GHs and HC IVs in central Uganda	
No.	Question/instruction
	Please tell us about yourself.
	Tell us about your responsibility towards the doctors working in public GHs and HC IVs.
	What are your views on CPD for doctors working in public GHs and HC IVs; the benefits, the concerns?
	What are your thoughts on the role of CPD for doctors? Currently? Potentially? in public GHs and HC IVs or primary health care of Uganda?
	What do you think are the issues in implementing CPD for doctors working in public GHs and HC IVs; strengths, opportunities, barriers, threats?
	What are your views on appropriate CPD for doctors working in public GHs and HC IVs; relevant inputs, and delivery process?
	What other aspects of the health care system are critical to the establishment of effective CPD for doctors working in public GHs and HC IVs?

CPD; continuing professional development, GHs; general hospitals, HC IVs; health center IV (HC IV)

**Data analysis:** manual descriptive thematic analysis [16] using a deductive approach was used. In a step-by-step process, the interviews were recorded, transcribed, and numbered. The responses were then coded and pooled to protect the confidentiality of participants. The interviews were transcribed verbatim by the PI and the research assistant. The PI transcribed seven of the interviews to gain a better understanding of the participants´ responses. The research team read the transcripts and identified themes and sub-themes related to CPD practices, facilitators, benefits, challenges and suggestions. Codes were developed independently by researchers reading through the transcript line by line, making labels and comments on margins. Each transcript was independently coded by two different members of the research team. This process was completed for the first six transcripts. The final set of codes was agreed upon by the research team meeting and discussing the codes and their decisions. The codes were defined and then grouped into categories to form a working analytical framework. Abbreviation letters were assigned to the different categories and codes with numbers for identification. The codes from this framework were then used to index the other transcripts. Data from each transcript was summarised by category. The research team met regularly to discuss impressions and ideas emerging from the data, their individual and collective interpretation of the data and to reach consensus on coding. The verbatim is reported in quotations and is italicized.

**Ethical considerations:** ethical clearance was obtained from the Makerere University School of Medicine Research and Ethics Committee (#REC REF 2019-126) and the Uganda National Council for Science and Technology (HS1170ES). The participants were contacted by telephone before a consent form was sent or delivered to them requesting their consent to participate. Written or verbal voluntary informed consent was obtained from participants before their interviews.

## Results

A total of 10 interviews were conducted and summarised when no new information was emerging and the study had reached saturation. Table 2 presents the description of the study participants. The stakeholder views were grouped into five themes; current CPD practices, facilitators, benefits, challenges, and suggestions. Table 3 summarises the subthemes that emerged from each theme according to the interviews.

**Table 2:** description of study participants

Participant	Age (years)	Gender	Duration of responsibility (years)	Nature of responsibility
P01	59	Male	15	Uganda Medical and Dental Practitioners Council
P02	50	Male	10	Family physician and District Health Officer
P03	51	Male	2	District Health Officer
P04	40	Male	2	Senior Medical Officer, Ministry of Health Uganda
P05	52	Male	2	Medical Superintendent
P06	34	Male	2	Uganda Medical Association, CPD committee Central Uganda
P07	45	Male	5	Family physician and Medical Officer Special Grade
P08	45	Male	1	Clinical Services Directorate, Ministry of Health
P09	56	Male	4	Family physician and Commissioner, Ministry of Health
P10	58	Male	5	Family physician and District Health Officer

**Table 3:** themes and sub-themes for views of stakeholders on CPD for doctors in the district health system, central Uganda

Themes	Subthemes
Current CPD practices	Trainings (physical and virtual)
	Mentorship and apprenticeship
	Support supervision
	Quality improvement projects
CPD facilitators	Internet services
	Grants
	Health facility managers
	Acknowledgement of facility-based CPD providers
	Harmonization of regional CPD guidelines and committees
CPD benefits	Motivation
	Updates knowledge
	Teamwork
	Renewal of annual practicing license
CPD challenges	Workload
	Allowances
	Access
	Documentation
	Mindset
	Structure of the public health care system
	Quality of CPD
	Sustainability
CPD suggestions	Regular training needs analysis
	Collaboration of district and regional health teams
	Monitoring
	Continuous support supervision and mentorship
	Stricter district administrators and councils on CPD
	Development of localized e-CPD platforms
	Regional CPD resource centers
	Shift of CPD responsibility

continuing professional development (CPD)

### Theme 1: current CPD practices

This theme talks about the various CPD activities that the doctors in the DHS take on in their workstations.

**Trainings:** participants described the existence of both physical and virtual CPD training2.. ‘*We have been learning together after that we demonstrate. Other demonstrations are practical for example resuscitating a baby, we use the dolls*.´ (P05) Participants reported an increase in online sessions, with the help of partnerships, following the COVID-19 era. '*…we have partnered with the World Commission of Education Alliance (WCEA) to provide CPD, you can access that portal and see what is happening, this is partnered with WHO to provide CPD to, especially rural places*.´ (P01)

**Mentorship and apprenticeship:** participants made a description of junior doctors being intentionally paired with existing senior doctors for purposes of continued mentorship in the work environment and beyond. '*…we do mentorships; you find that a doctor is attached to maybe two other junior doctors…*´ (P07) In some situations, apprenticeship was described where the doctors that have special training needs, for instance, surgical skills are paired with those that are experienced for continued learning. '*We pass on skills to those, if someone has better skills, he helps others who don´t have that particular skill*.´ (P05)

**Support supervision:** support supervision by the supervising facilities was highlighted as an avenue for CPD among the doctors in the DHS. '*What we were mainly targeting in the support supervisions is preventive medicine because it is cheaper and easier, for instance during the COVID-19 time, our role was to emphasize the value of SOPs in the management of community COVID-19*.´ (P07) '*…we do support supervision to those lower facilities…*´ (P09)

**Quality improvement projects:** the quality improvement projects encourage practical interventions for local problems and at the end of the project, the positive outcomes greatly impact the continued learning of the doctors involved. '*This quality improvement approach is intended to improve certain indicators; it is this same doctor who is introducing the change that … continues to learn from what they have introduced …*´ (P09)

### Theme 2: CPD facilitators

This theme relates to the existence of local policies, initiatives, and assets that make it possible for primary care doctors to ensure that they interface with the available CPD opportunities.

**Internet services:** the availability of internet services was identified as a strong CPD facilitator from the interviews. This made it possible for doctors to carry on with CPD sessions via Zoom, join other CPD platforms, and think of more innovations that foster CPD. '*…we can also be on Zoom with other health facilities and share information, we have facilities for Zoom, it was offered by Mildmay. They gave us a big television, plus they connected us to the internet*.´ (P05)

**Grants:** the Ministry of Health and its implementing partners were reported to provide funding for support supervision and the development of regional CPD resource centers. The resource centers are to serve the doctors and all other cadres in the lower-level health facilities supervised. '*So the partnership we made with USAID was to make regional hospitals centers for provision of CPD especially where there is little capacity*.´ (P01) '*…currently there is a funding agency from Korea, they support the regional referral hospitals to monitor and supervise health facilities especially HC IVs, so for us, we are using that window, … If it is said, Advanced Life Support, the CPD we want, we tell them and slot it on the day when they will come..*.´ (P03)

**Health facility managers:** participants noted that in some of the public primary care facilities, the persons in charge advocate for CPD while others may not care. Their active advocacy was described as ensuring that time and money are allocated for CPD activities. Some were charged with CPD activities directly, for instance, mentorship, conducting CPD sessions for fellow doctors, and spearheading quality improvement projects. '*In HC IVs where they have a budget, if you get a good focal person …can convince the administration to allocate … incentives for continuing medical education sessions e.g. a soda. …if they have PHC allowances… they use it as an opportunity to get some refreshments… on that day when they have the CPDs or … as an allowance…*´ (P02) '*…there are in-charges in the know and would allow time the staff for the CPDs..*.´ (P10).

**Acknowledgement of health facility-based CPD providers:** the participants noted that recently the UMDPC advertised for facility-based CPD providers to register with it for recognition. This has partially improved the facilitation and information flow to the UMDPC about CPD activities especially when the doctors need proof of CPD as they renew their annual practicing licenses. '*…we allowed the smaller units at the district, at the HC IVs if they have more than two or three doctors to apply as CPD providers and they are accredited ´*(P01).

**Harmonization of regional CPD guidelines and committees:** the medical councils of the different countries in the East African region are working together and have harmonized their CPD requirements, therefore when the doctors receive CPD points in the region they are recognized. '*CPD became an East African item, among the things we have harmonized… so there is an opportunity as East Africans to grow the concept so that we have mutual recognition of CPD across the region*.´ (P01). It was also reported that some of the recent guidelines by the UMDPC are primarily focused on CPD opportunities. For instance, the guideline on medical camps emphasizes local leadership of a doctor so that at the end of the camp continued learning of both skills and knowledge has happened. ‘*… we have put a requirement in the medical camp guidelines that a local doctor should be in charge of the camp …. the main thing to the council, is there skills transfer in that camp…?*’ (P01)

### Theme 3: CPD benefits

This theme speaks to the observed attributes that come with primary care doctors involving themselves actively in CPD.

**Motivation:** the doctors are described as motivated to put into practice what they have learned and, in the process, they keep themselves up to date. '*When medical officers are trained, they are motivated because they have skills…*´ (P04).

**Updates knowledge:** most participants mentioned CPD as an opportunity for doctors to update themselves with new knowledge in the field of medicine which generally impacts the way they manage patients. '*CPD …helps update health workers about new developments in medicine, especially in the areas of HIV, we have now new vaccines and other technologies*.´ (P02) ' *…it´s a moment of sharing new and old information and it gives us better management of our patients and it keeps us updated of every new information*.´ (P05)

**Teamwork:** the doctors in the primary care facilities work closely with all the other cadres and CPD activities are not an exception. Participants have described this as an opportunity to strengthen teamwork. '*On the non-technical bit, it also helps to create teamwork and cohesion, …it brings different departments together, from maternity to, the general ward and they can know each other*.´ (P02) '*…we are only two medical officers in the HC IV so even other people are involved in that CPD*.´ (P05)

**Renewal of annual practicing license:** the participants all acknowledged the fact that evidence of CPD makes your renewal process of the annual practicing license much faster. '*…everybody knows that CPD is required, there are no longer people who say we didn´t know give us another year*.´ (P01) '*…when you are going to renew your license, they ask you for those CPD things…*´ (P05)

### Theme 4: CPD challenges

This theme describes the hurdles experienced by primary care doctors in trying to ensure that they participate effectively in CPD activities.

**Workload:** the commonest challenge described by the participants was the doctors´ workload at the health facilities that would get in the way of the scheduled CPD. '*…other competing activities… like campaigns, studies or assessments coming from the center (Ministry of Health) they disrupt their organization leaving a skeletal staff that cannot form an adequate quorum for CPD*.´ (P02) '*…the HC IV is becoming too big, … the challenge is that sometimes you can schedule a CPD but when you reach the facility you find the whole place clogged then you decide to start with the patients. You can then miss the CPD so the workload is one of the biggest challenges…*´ (P10)

**Allowances:** most participants described scenarios that involved providing CPD presenters and participants with some form of allowance which is commonly not available. This in the long run reduces on the frequency and quality of the CPD sessions. '*…we do not have any facilitation to the presenters in terms of data refund yet they are sharing knowledge …at the end of the day they have to meet their expenses of data and time to prepare*.´ (P06) '*Motivation among the doctors, like at least if there are refreshments, it would attract most of the participants and they would get energized internally*.´ (P07)

**Access:** the participants described access in several dimensions; small e-platforms to accommodate the doctors, access to internet and electricity, availability of physical space by the medical association for regular CPDs, and access to quality senior presenters or mentors as a whole. '*…the challenge is getting the people to present and also the platform to use, the zoom, you try to follow a link from one organization then you find that during the session it only accommodates 100 then you run to another organization and you find that the person-in-charge is offline. People get stranded and you spend an hour or so trying to get an alternative*.´ (P06) '*some specialists… do not want to conduct zoom sessions because people do not pay attention and they would want a physical space, …we do not have the physical space…*´ (P06) '*…we have limited access to internet connectivity and sometimes even power so it would be very difficult for you to have doctors in the rural settings attend …*´ (P08)

**Documentation:** participants identified documentation of CPDs as a challenge that affected the process of renewal of the practicing licenses at the UMDPC. The support supervision teams were equally affected by the lack of documentation on CPD because in their absence it was unclear what the doctors covered. ‘*The CPD which they do is not submitted so sometimes we get problems with some congestion because we have put the online licensing and requires CPD but because the providers have not provided the CPD record when the doctors go to update and print, they find that their CPD record has not come*.´ (P01) '*When we are doing support supervision, we found that they are not documenting …if you have no evidence, it reflects that you are not doing CPD*.´ (P07)

**Mindset:** some of the participants mentioned the poor mindset towards CPD among some of the doctors and health facility managers. They do not value CPD as an important entity that improves service delivery and therefore make no efforts to ensure that it is planned. '*…staff overlook the importance of CPD. Even the managers (in charge) themselves do not view CPD as an important aspect in improving service delivery, they look at it as a routine activity because when assessors come for service assessment, they tick it off as an activity that happens*.´ (P02) '*The biggest barrier is mindset…, many of us tend to underrate services or knowledge and information outside our general practice …so when you bring them up in leadership… few.. appreciate the benefit that such a skill or knowledge brings…*´ (P08)

**Structure of the public health care system:** participants identified a challenge with the current structure of the public health care system which in turn affects the stewardship of CPD for the doctors in the public primary care health facilities. '*There is… a challenge in the way the health care system is arranged in the country, you find that structurally general hospitals and lower-level health facilities are subscribed or subordinate to the referral hospital but administratively these are two different entities. The general hospitals and lower-level health facilities belong to the local government while the regional referral hospitals belong to the Ministry of Health so sometimes the leadership challenge becomes a limitation to effective supervision*.´ (P08) '*…the challenge is the structure of the system… the medical officers are appraised by...the health facility in-charge so when they come to the district it is just an endorsement*.´ (P10)

**Quality of CPD:** participants highlighted the fact that there is no clear measure of the quality of the CPDs that are being conducted and which competencies are being affected. '*Practicability, how do you know that the CPD is improving competencies…, apart from getting the 48 hours?*´ (P01) '*The quality of CPD may not be verified to see if it is useful to the practitioner…*´ (P10)

**Sustainability:** the UMDPC plans to maintain a constantly renewed list of CPD providers in order to check on quality. The initial registration of CPD providers was free, there is no guarantee that this register will be maintained if the UMDPC starts levying regular registration fees. '*…some agencies funded efforts to make the CPD guidelines, we accredited the CPD providers free of charge for two years, the assumption was that after people understanding the importance, they will pay…*´ (P01)

### Theme 5: CPD suggestions

This theme echoes the different views held by the stakeholders on what could be done to upgrade current CPD initiatives.

**Regular training needs analysis:** participants observed that regular CPDs for the doctors are necessary and should be informed by CPD training needs analysis so that they are relevant. ' *Training to target the doctors on things that they have suggested in form of transferring skills, for instance in workshops*.´ (P07) '*The CPD issues are raised by us… we discuss a certain topic and we go to work*.´ (P03)

**Collaboration:** participants suggested more active involvement in CPD of the district health leadership which would in turn utilize the resources at the regional level. '*…we would get facilitators not from within the facility, …from the district, …not from their local environment… I also think for the doctors it would be better, if the DHOs office has some facilitation… we would get …a consultant from the regional referral or anybody whom we think has the knowledge to come and deliver…*´ (P02) Other suggested collaborations on CPD included; the Uganda Medical Association (UMA) and the accredited CPD providers, the academic institutions with primary care facilities, and UMDPC with specialist associations. '*…if possible institutions which have e-platforms already, for example, medical schools would support UMDPC and UMA*.´ (P10) '*…the CPD providers list, it is quite large so going forward one of the things we have to strengthen is the collaboration …to ensure continuity and regularisation of CPD*.´ (P06) '*…the people who are in academia or… senior consultants… in our region... to share with us how they manage cases …there is a way these specialists handle their patients that is different…, this could improve the delivery of the CPD*.´ (P03)

**Monitoring:** the doctors report participating in various CPD activities that need to be monitored for feedback on the impact of the quality of primary care services. '*…research to see how the CPDs are improving …outcomes, …care to patients as a result of CPD? So does it show there are less accidents, less inquiries at the council, less medical errors, does CPD cater for some soft skills…*´ (P01) '*Follow up mechanism on those that have participated in CPD to see whether there is an impact or some improvement needed*.´ (P02)

**Continuous support supervision and mentorship:** in some facilities, the current supervision and mentorship reported by participants is in reaction to an event. The suggestion is to move from reactionary to continuous support supervision and mentorship for a better impact on the quality of primary care services offered. '*The regional referrals are not very effective with support supervision, it happens if we have a problem, for example, lots of maternal deaths and we think there is a problem in the management of… post-partum haemorrhage, then we invite the consultant and invite all the medical officers to a convenient place to take them through*´. (P02)

**Stricter district administrators and councils on CPD:** some participants suggested that CPD should be one of the requirements for the doctor´s performance appraisal at the district level. It was also described as an obstacle to have the medical council emphasizing CPD while the other health cadres with whom they work in the primary care team have no strict CPD requirement from their respective councils. '*…we, the managers at the district level, need to pay thorough attention when we are assessing or appraising the medical officer*.´ (P02) '*We started with CPD as a medical council unfortunately other councils are not moving on the same pace however, we later agreed ….to make them guidelines because the CPD… will not be useful if you are doing it alone*.´ (P01)

**Development of local e-CPD platforms:** it was noted that CPD is moving from physical to virtual space, and participants suggested further development of localized e-platforms, e-granaries, teleradiology, and Zoom licenses purchased by DHS for collective sessions of their primary care facilities. '*…development of a platform where… CPD materials can be placed, given that all doctors cannot be there at the same time... someone can read at their pace…, and do a quiz and once they score the set mark they can be awarded their CPDs automatically*.´ (P06) '*One would be encouraging them to have zoom licenses at the different units, such that it´s done at a central unit whereby most of them log in and they could have different views from the different health workers and they could be able to share views from different facilities too and improve on their knowledge and skills* (P07) '*…other areas to look at that have advanced technology and can be applicable on phone, for example, on tele radiology because a medical officer who is somewhere in Bwera if he can study and apply radiology at his level using a Radiologist who is in Kampala using tele radiology, so even telemedicine, those are areas we can look into* ´ (P08).

**Regional CPD resource centers:** participants suggested more focus on the idea of regional CPD resource centers. These were first suggested by the specialist organizations for continued learning of members and now participants in this study recommend borrowing the idea for the doctors in the DHS. '*So the partnership we made with USAID was to make regional hospitals as centers for provision of CPD especially where there is little capacity. So we have piloted in Jinja, Lira, Mbarara and Mbale and the funders are to make those CPD sites supportive of the districts and other programs that are in their regions. So we are yet to role that one out and I think it will help especially those areas where there are few doctors…. so we think these CPD areas will be having a resource center, the regional referral hospitals have big capacity there are so many people there who can provide CPD so it will be useful at that level*.´ (P01) '*We have partnered with specialist associations to improve the quality of CPDs which are being provided…, that is why we are talking about those regional centers such that CPDs are complimented with practicum…*´ (P01) '*We need to have good documentation because …there is a lot of CPD, there are so many people who are coming there and we are trying to engage many partners on board, but some of the things we need to add are involvement of the seniors, creating a platform for those who might be willing to share knowledge. We also think that the higher institutions of learning like the colleges of health sciences can always support, …collaborate with us and ensure that at least we have those reassured CPD events to ensure continuity but also ensure that they are regular*. (P06)

**Shift of CPD responsibility:** the current responsibility to ensure the CPD of the doctors is organized partially lies with the UMDPC and it was suggested that with adequate empowerment, this responsibility should entirely shift to the individual doctor. '*So we are looking at that level where people will really find that it is my responsibility it is not a council requirement… to improve, because eventually, it affects your status, it affects your money´. (P01)*

## Discussion

The purpose of this study was to explore stakeholders´ views on CPD for doctors working in the DHS in central Uganda. The participants´ views highlighted the current CPD practices, facilitators, benefits, challenges, and suggestions of CPD that cater to the present context. The CPD practices included; training, mentorship and apprenticeship, support supervision, and quality improvement projects. The facilitators were; internet services, grants, health facility managers, facility-based CPD providers, and regional CPD guidelines. The main benefits were; motivation, knowledge updates, teamwork, and renewal of practicing licenses. Key challenges were; workload, allowances, access, documentation, mindset, quality of CPD, structure of the public health system, and sustainability. The following were suggestions; training needs analysis, collaboration, monitoring, e-CPD platforms, CPD resource centers, and individual CPD responsibility.

All participants were male which reflects their dominance in decision-making positions in the primary care settings of this region. This may also have implications for missed views on CPD with a female perspective on the practices, facilitators, challenges, and suggestions. The importance of gender was emphasized in a study that highlighted deterrents to women´s participation in CPD [17]. The current CPD practices are molded by feasibility, affordability, and trends. Some of the practices are now more commonly employed than others because of the trends that are shifting from the analog to the digital era. The COVID-19 pandemic also further made health systems reflect on how monitored CPD could be achieved, this modified and magnified the possibilities of keeping doctors up-to-date with knowledge and skills [18,19]. The CPD facilitators highlighted in this study are majorly controlled by institutions responsible for the doctor´s professional performance. This could be explained by the fact that the participants are from these institutions with CPD as part of their core responsibilities. Meanwhile, the CPD benefits identified in this study are to both the individual doctors and the primary care system. This may be because the participants are also practitioners and are therefore personally interested in a functional CPD system.

On the other hand, the CPD challenges are a reflection of the primary care context in this region therefore they need to be taken into account with potential CPD innovations. The participants´ proposed suggestions taken into consideration the current developments in knowledge and skills acquisition. The responsibility of the suggestions made majorly lies with the different institutions responsible for the professional well-being of doctors working in the DHS. The institutions include the district health office, UMDPC, UMA (central branch), and MoH. The suggestions are crowned with the hope that over time the individual doctors will develop a culture of assessing and dealing with their own CPD needs. This is because of the continued availability of local and international resources to improve one´s knowledge and skills in several fields. The Indian health system also hopes doctors to be responsible for their CPD in the future [9].

The views on CPD in this study are a reflection of context, similar settings have comparable findings [7,8,15]. For instance, it has been noted elsewhere that the primary healthcare leadership has been mainly male-dominated [20]. Considering CPD practices, virtual training sessions are the most current and their popularity was gained during the COVID-19 era [18]. Despite the challenges that came with virtual CPD sessions, institutions embraced it and are continuously working on the gaps. Quality improvement projects as a CPD method are very practical and have a guide [21], they were officially introduced into the Ugandan health system by the Ministry of Health and its implementation partners. Clinical mentorship [22] and apprenticeship [23] are also in use elsewhere as a CPD practice and the common gap with this practice in some settings is a lack of an intentional structure. Some of the CPD facilitators identified in this study have also been identified in other settings, for instance, internet services, grants, and CPD guidelines [7,8]. This points to a universal need for CPD and therefore a need to work in teams to share resources. The availability of a coaching expert for clinical procedures has also been identified in other settings as a CPD facilitator [24].

In this study setting this idea could be explored for the future under the facility-based CPD providers. The benefits of CPD for doctors depend on the responsibility of the study participants. Studies whose participants are doctors in practice highlighted more personal benefits [5] compared to doctors in leadership positions in a health system [25]. Similarly, the findings on CPD benefits in this study are inclined to investment in the available human resources. Some of the challenges have also been highlighted in previous international research [5,7] while others like the structure of the public health system are unique to context. For instance, a study in Ireland found that slow internet, workload, and relevant CPD activities were challenges to CPD for doctors [5]. The challenges of time and cost commonly identified in other research on CPD have been implied under workload and allowances in this study. The suggestions in the study trigger continued reflection on CPD in the DHS to ensure the best fit for the context given the limited resources. The reflection needs to take into consideration previous proposals on the CPD system that should be grounded in the everyday workplace, integrated into the health care system, oriented to patient outcomes, team-based, employing strategies of quality improvement, and collective responsibility of physicians CPD providers and regulators.

Overall, findings from this study have key implications for practice. For example, the innovative use of the internet to improve access to CPD information impacts practice. These findings do augment what has been previously documented in the literature but also offer some more empirical evidence regarding the need for CPD in low-resource settings in such a way that the key health leadership views are taken into consideration. We do recommend more studies in different settings with a variety of key stakeholders that drive the CPD agenda for primary care doctors. Further research specifically focusing on what they view as competencies improved by effective CPD is recommended.

**Strengths and limitations of the study:** the strengths of this study are based on interviews with a cross-sectional sample of key persons that influence the performance and CPD needs of primary care doctors. Since participants were selected to represent stakeholders from both central and local governing bodies, the study is likely to have identified the common practices, benefits, challenges, and realistic adjustments of CPD. Like in many qualitative studies, we relied entirely on the participants´ opinions which would not necessarily be similar in other settings. However, the study provides key information upon which other studies can build.

## Conclusion

This study emphasizes the importance of CPD for primary care doctors in the delivery of primary health care. Therefore, continued collaborative efforts to work on the CPD challenges are hoped to improve primary care services in the DHS. Solutions to the challenges or implementation of the suggested solutions is a collective responsibility that may call for teamwork from both the stakeholders and primary care doctors.

### What is known about this topic

- *Continuing professional development is a key component of quality improvement in health;*
- *Continuing professional development reinforces professional lifelong learning*.

### What this study adds

- *The healthcare leaders are interested in making the continuing professional development experience of their primary healthcare teams worthwhile;*
- *The COVID-19 era made the stakeholders and the doctors think about more ways of using the internet to augment continuing professional development*.
